# MiR-92b-3p Inhibits Proliferation of HER2-Positive Breast Cancer Cell by Targeting circCDYL

**DOI:** 10.3389/fcell.2021.707049

**Published:** 2021-07-29

**Authors:** Gehao Liang, Yun Ling, Qun Lin, Yu Shi, Qing Luo, Yinghuan Cen, Maryam Mehrpour, Ahmed Hamai, Jun Li, Chang Gong

**Affiliations:** ^1^Breast Tumor Center, Sun Yat-sen Memorial Hospital, Sun Yat-sen University, Guangzhou, China; ^2^Department of Breast Oncology, Sun Yat-sen University Cancer Center, Sun Yat-sen University, Guangzhou, China; ^3^Department of Breast Surgery, The Second Affiliated Hospital, Guangzhou Medical University, Guangzhou, China; ^4^Department of Biochemistry, Zhongshan School of Medicine, Sun Yat-sen University, Guangzhou, China; ^5^Institut Necker-Enfants Malades (INEM), Inserm U1151-CNRS UMR 8253, Paris, France

**Keywords:** MiR-92b-3p, circCDYL, cell proliferation, HER2-positive breast cancer, RNA induced silencing complex

## Abstract

**Objectives:**

Circular RNA (circRNA) is a novel class of RNA, which exhibits powerful biological function in regulating cellular fate of various tumors. Previously, we had demonstrated that over-expression of circRNA circCDYL promoted progression of HER2-negative (HER2^–^) breast cancer *via* miR-1275-ULK1/ATG7-autophagic axis. However, the role of circCDYL in HER2-positive (HER2^+^) breast cancer, in particular its role in modulating cell proliferation, one of the most important characteristics of cellular fate, is unclear.

**Materials and methods:**

qRT-PCR and *in situ* hybridization analyses were performed to examine the expression of circCDYL and miR-92b-3p in breast cancer tissues or cell lines. The biological function of circCDYL and miR-92b-3p were assessed by plate colony formation and cell viability assays and orthotopic animal models. In mechanistic study, circRNAs pull-down, RNA immunoprecipitation, dual luciferase report, western blot, immunohistochemical and immunofluorescence staining assays were performed.

**Results:**

CircCDYL was high-expressed in HER2^+^ breast cancer tissue, similar with that in HER2^–^ breast cancer tissue. Silencing HER2 gene had no effect on expression of circCDYL in HER2^+^ breast cancer cells. Over-expression of circCDYL promoted proliferation of HER2^+^ breast cancer cells but not through miR-1275-ULK1/ATG7-autophagic axis. CircRNA pull down and miRNA deep-sequencing demonstrated the binding of miR-92b-3p and circCDYL. Interestingly, circCDYL did not act as miR-92b-3p sponge, but was degraded in miR-92b-3p-dependent silencing manner. Clinically, expression of circCDYL and miR-92b-3p was associated with clinical outcome of HER2^+^ breast cancer patients.

**Conclusion:**

MiR-92b-3p-dependent cleavage of circCDYL was an essential mechanism in regulating cell proliferation of HER2^+^ breast cancer cells. CircCDYL was proved to be a potential therapeutic target for HER2^+^ breast cancer, and both circCDYL and miR-92b-3p might be potential biomarkers in predicting clinical outcome of HER2^+^ breast cancer patients.

## Introduction

Breast cancer (BC) has become the most common malignant tumor and leading cause of death by tumor among female worldwide (Bray et al., 2018). Approximately 15–20% of these BC patients exhibits over-expression/amplification of human epidermal growth factor receptor 2 (HER2), and this BC subtype is named as HER2-positive (HER2^+^) breast cancer. Although anti-HER2 therapy together with operation and chemotherapy contribute to a favorable clinical outcome of HER2^+^ BC patients, around 10% HER2^+^ patients still suffer recurrence or metastasis with the reasons unknown (DeSantis et al., 2019). The underlying mechanisms, especially non-coding RNA, in progression of HER2^+^ BC progression are not elucidated clearly.

Circular RNAs (circRNAs), a novel class of RNAs with covalently closed loop, are stable and abundant in mammalian cells (Yu and Kuo, 2019). Importantly, circRNAs exhibit disease-specific and disease progression-specific characteristics, indicating that circRNAs exhibit a biomarker potential in early diagnosis and predicting prognosis of human disease (Jahani et al., 2020). Several researches demonstrated that circRNAs could act as miRNA sponges (Memczak et al., 2013; Yang et al., 2019; Zhou C. et al., 2020), and certain circRNAs with open reading frame (ORF) and internal ribosome entry site (IREs) acted as templates for protein translation (Legnini et al., 2017; Yang et al., 2018; Zhang et al., 2018), while the circRNAs located in nucleus regulated expression of certain genes by interacting with RNA polymerase II (Zhang et al., 2013; Li et al., 2015). In addition, circRNAs display powerful biological functions such as proliferation, migration, invasion and chemotherapy resistance in HER2-negative (HER2^–^) subtype of breast cancer (Yang et al., 2019; Zhou Y. et al., 2020; Wang L. et al., 2021). Nevertheless, the role of circRNAs in regulating the cellular fate of HER2^+^ BC cells is rarely elucidated.

Our previous study identified an autophagy-associated circRNA circCDYL, which promoted autophagosome formation and proliferation of HER2^–^ breast cancer cells by sponging miR-1275 (Liang et al., 2020). However, the roles of circCDYL, especially cellular fate including proliferation and autophagy in HER2^+^ BC, has not been investigated. In this study, circCDYL was found to promote the cell proliferation as well, but exhibited little effect on autophagic level in HER2^+^ BC cells. Mechanism study demonstrated that little miR-1275 was found to bind circCDYL, as miR-1275 rarely expressed in HER2^+^ BC cell lines. By miRNA sequencing, miR-92b-3p was screened out to bind with circCDYL in HER2^+^ BC cell lines. Interestingly, circCDYL did not act as miR-92b-3p sponge, but was degraded in a miR-92b-3p dependent silencing manner.

## Materials and Methods

### Cell Culture and Treatment

Normal human mammary epithelial cells (MCF-10A), HER2-negative BC cell lines (MCF-7, MDA-MB-231, and ZR75-1) and HER2-positive BC cell lines (AU565, MDA-MB-361, SK-BR-3, and BT474) were used in this study. All these cell lines were cultured as ATCC recommended. For transient transfection, 125 ng circCDYL over-expressing plasmid or 3 pmol siRNA (siRNA sequence shown in Supplementary Table 1) was added to cells with lipofectamine 3000 (Invitrogen, United States). Total RNA or protein was extracted 48 h after transfection.

### Plate Colony Formation Assay

The SK-BR-3 or BT474 cells (3 × 10^3^) were seeded in 6-well plates and were cultured for 3 weeks. The colonies were fixed with 4% formaldehyde for 10 min and stained with 1% crystal violet for 30 min. Image J software was used to calculate the colonies in each group.

### Cell Counting Kit-8 Assay

The SK-BR-3 or BT474 cells (3 × 10^3^) after treatment were seeded in 96-well plates. After 5 days, cellular viability of each group was determined by Cell Counting Kit-8 (Tongren, Japan).

### Cell Counting Assay

Total of 5 × 10^4^ SK-BR-3 or BT474 cells after treatment were cultured in 12-well plates. In continuous 5 days, cells were digested by trypsin, and re-suspended. Then cell suspension was added into cell-counting plate (Ruiyu Bio-science, China), and the cell number was calculated by cell counter (Countstar Bio-tech, IC100, China).

### RNA Isolation and qRT-PCR

The total RNA of cell lines was collected by TRIzol reagent (Invitrogen, United States). The RNA was reverse transcribed to cDNA using RT SuperMix (Vazyme Biotech, China) according to the manufacturer’s protocol. The real-time quantitative polymerase chain reaction (qRT-PCR) was performed by SYBR qRT-PCR Master Mix (Vazyme Biotech, China) on LightCycler 480 II system (Roche, Switzerland). The sequence of primers in this study were shown in Supplementary Table 2.

### Western Blot Analysis

Cells were lysed in RIPA lysis buffer (BCA) kit (Lot 30342, Cwbio, China) was used to detect the concentration of protein. In electrophoresis process, 12% SDS-PAGE was used. The protein in the gels were transferred to PVDF membrane, and the membrane was incubated with 5% non-fat milk at room temperature. Primary antibody anti-β-actin (Cell Signaling Technology, 1:1,000), anti-PI3K (Cell Signaling Technology, 1:1,000), anti-p-PI3K (Cell Signaling Technology, 1:1,000), anti-AKT (Cell Signaling Technology, 1:1,000), anti-p-AKT (Cell Signaling Technology, 1:1,000) and anti-LC3 (Sigma-Aldrich, 1:1,000) were, respectively, added to the membrane and incubated at 4°C overnight. Secondary antibody (Cell Signaling Technology, 1:3,000) was added and incubated for 2 h at room temperature. Finally, the blots were detected by enhanced chemoluminescence kit (P90719, Millipore, United States) under Image Lab Software.

### MiRNA Pull-Down Assay

MiRNA pulldown experiment was processed according to previous publication. Briefly, SK-BR-3 cells were transfected with biotinylated miR-92b-3p mimic. After 24 h, transfected cells were harvested and fixed with formaldehyde for 30 min. Then the cells were lysed by co-IP buffer. C1 streptavid in magnetic beads was added to the mixture. Finally, total RNA was extracted as described and followed by qRT-PCR detection of circCDYL.

### miRNAs Deep-Sequencing

RNA samples enriched by circCDYL probes in circRNAs pull-down assay was sent to Kangchen Bio-tech Company (Shanghai, China) for miRNA deep-sequencing and subsequent analysis. The raw data of miRNA deep-sequencing was uploaded to GEO database (GSE174541).

### *In situ* Hybridization and Fluorescence *in situ* Hybridization

*In situ* hybridization (ISH) was performed to detect the expression of circCDYL and miR-92b-3p in paraffin-embedded sections from BC tissues or animal tumors. Briefly, the sections were digested with pepsin after dewaxing and rehydration, and hybridized with the digoxin-labeled circCDYL probe at 37°C overnight. Then the sections were incubated with anti-digoxin antibody overnight at 4°C and were stained with nitro blue tetrazolium/5-bromo-4-chloro-3-indolylphosphate. The staining scores were determined based on both the intensity and proportion of circCDYL. Total score = Σproportion × intensity. Intensity was recorded as 0 (no staining), 1 (light purple), 2 (purple blue), or 3 (dark purple). As for fluorescence *in situ* hybridization (FISH), Cy3-labeled probe for circCDYL and FAM-labeled probe for miR-92b-3p were used. These probes were designed and synthesized by Synbio-Tech Company (Guangzhou, China). Briefly, cells were cultured in a glass-bottom dish overnight and incubated with pre-hybridization solution at room temperature for 30 min. 20 μM of probes in hybridization solution was added to dish and hybridized overnight. After washing by SSC (saline sodium citrate), the dishes were incubated with DAPI for 10 min. Finally, the dishes were covered with coverslip and observed by confocal microscope.

### CircRNA Pull-Down

CircRNA pull-down was performed as previously reported (Liang et al., 2020; Zhou C. et al., 2020). Briefly, SK-BR-3 was fixed with 1% formaldehyde and lysed by co-IP buffer and the cluster was sonicated. CircCDYL-specific and NC biotinylated probes were added to mixture to bind circCDYL. Next, C1 streptavidin magnetic beads was added to pull down circCDYL and circCDYL-binding RNA. Finally, total RNA was extracted from the magnetic beads and followed by qRT-PCR detection of circCDYL and miR-92b-3p.

### Dual Luciferase Reporter Assay

Full-length sequence of circCDYL was inserted into psiCHECK-2 vectors (Synbio-tech, China). psiCHECK-2 vectors carrying NC mimic or miR-92b-3p mimic were co-transfected to SK-BR-3 cells, respectively. Dual-luciferase reporter assay system (Vazyme, Nanjing, China) was performed to detect luciferase activity of the transfected cells after 48 h transfection.

### *In vivo* Breast Cancer Orthotopic Model

The breast cancer orthotopic model were performed in Forevergen Medical Corporation (Guangzhou, China), and all procedures were in accordance with the ethical guidelines of the institution. Briefly, we purchased 4-week-old female Balb/c nude mice from Nanjing Biomedical Research Institute of Nanjing University (Nanjing, China). SK-BR-3 cells (1 × 10^7^) transduced with sh-NC or sh-circCDYL lentivirus were injected into the fourth left mammary fat pads of the nude mice (*n* = 6/group). The tumor growth of each group was recorded. After 44 days since tumor cell plantation, the mice were executed, and the tumor was made into paraffin-embedded sections.

### Patient and Clinical Database

In this study, 50 HER2^+^ BC patients and 70 HER2^–^ BC patients from Sun Yat-sen Memorial Hospital (SYSMH) were enrolled. Enrolled patients were firstly diagnosed without any distant metastasis between 1st January 2010 and 31th December 2017. Paraffin-embedded BC tissue sections were collected for *in situ* hybridization (ISH). Clinicopathological material, such as age, molecular subtype, stage, survival, was collected and analyzed.

### Statistical Analysis

GraphPad Prism 5 software was used for statistical analyses in this study. Student’s *t*-test was performed to test statistical differences between two subgroups. *X*^2^-test was applied to analyze the correlations between circCDYL expression and clinicopathological characterization of HER2^+^ BC patients. Survival analysis of HER2^+^ patients was evaluated by Kaplan–Meier plots and Log-rank tests. The univariate analyses were evaluated by Cox proportional hazards model. *P* < 0.05 was considered statistically significant.

## Results

### CircCDYL Expression in HER2 + Breast Cancer Is Similar With That in HER2^–^ Breast Cancer

**FIGURE 1:**
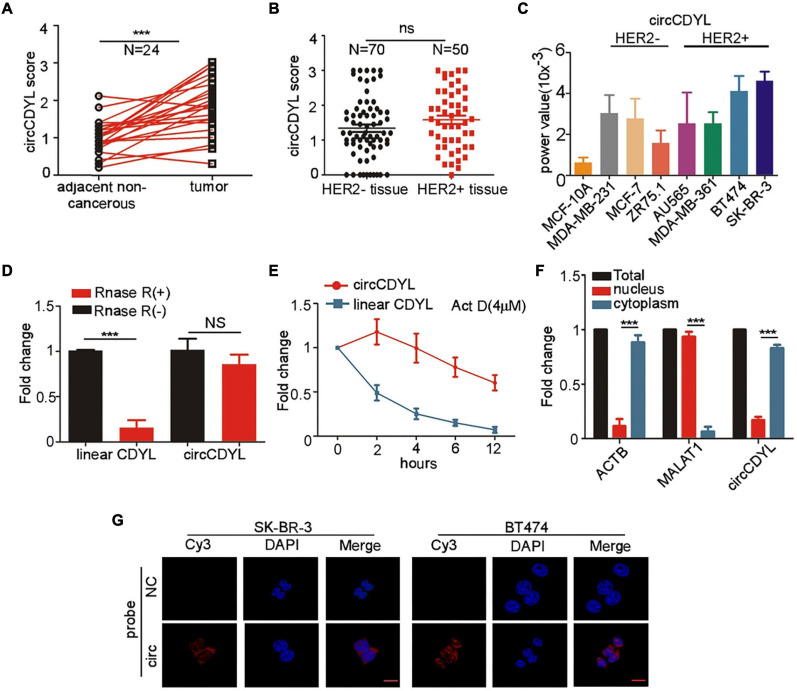
CircCDYL is high-expressed in HER2^+^ BC. **(A)** Comparison of circCDYL expression between HER2^+^ BC tissues and paired adjacent non-cancerous tissues by *ISH* (*n* = 24). **(B)** Expression of circCDYL in HER2^–^ BC tissues (*n* = 70) and HER2^+^ BC tissues (*n* = 50), as detected by *ISH*. **(C)** Expression of circCDYL in normal mammary epithelial cells (MCF-10A), HER2^–^ BC cell lines (MCF-7, MDA-MB-231, and ZR75-1) and HER2^+^ BC cell lines (AU565, MDA-MB-361, SK-BR-3, and BT474), as detected by qRT-PCR. **(D)** Linear CDYL and circCDYL before or after RNase R digestion was detected by qRT-PCR. **(E)** qRT-PCR detection of circCDYL and linear CDYL in SK-BR-3 cells treated with Actinomycin D (4 μM) at various times. **(F)** qPCR analysis of circCDYL in the cytoplasm and nuclear separated from SK-BR-3 cells. **(G)** Cellular location of circCDYL in SK-BR-3 and BT474 cells, as detected by FISH. All experiments above were done for at least three times. ***P < 0.005. Error bars indicate Standard Error of Mean (S.E.M).

To determine the expression of circCDYL in HER2^+^ breast cancer, 50 HER2^+^ patients and 70 HER2^–^ patients were enrolled. *ISH* analysis revealed that circCDYL was elevated up to 1.63-folds in tumor tissues, compared to adjacent normal tissues of HER2^+^ BC patients (*n* = 24) (Figure 1A). However, the expression level of circCDYL in HER2^+^ BC tissues was similar to that in HER2^–^ BC tissues (Figure 1B). In addition, the expression of circCDYL was detected by qRT-PCR in normal mammary epithelial cells (MCF-10A), HER2^–^ BC cell lines (MCF-7, MDA-MB-231, and ZR75-1) and HER2^+^ BC cell lines (AU565, MDA-MB-361, SK-BR-3, BT474). CircCDYL expression was obviously higher expressed in HER2^+^ BC cell lines than normal mammary epithelial cells, slightly higher than that in HER2^–^ BC cell lines (Figure 1C). In addition, silencing HER2 gene in SK-BR-3 cells and over-expression of HER2 gene in HER2^–^ BC cells (MCF- 7 and MDA-MB-231) had no effect on expression of circCDYL, indicating that HER2 gene do not regulate the expression circCDYL in HER2^+^ cell (Supplementary Figures 1A,B).

CircCDYL (chr6:4,858,880-4,925,679) is derived from exon 4 of gene Chromodomain Y Like (CDYL). We further examined the characteristic of circCDYL. As shown in Figures 1D,E, circCDYL was more stable than parent CDYL linear RNA after RNase R digestion and more stable than CDYL linear RNA in living cells after inhibiting transcription of SK-BR-3 cells by actinomycin D (Act D, a transcription inhibitor). Moreover, we found that circCDYL mainly located in cytoplasm, as detected by nuclear-cytoplasm separation experiments (Figure 1F) and FISH in SK-BR-3 and BT474 cells (Figure 1G).

### Over-Expression of circCDYL Promotes Cell Proliferation of HER2^+^ BC Cells

**FIGURE 2:**
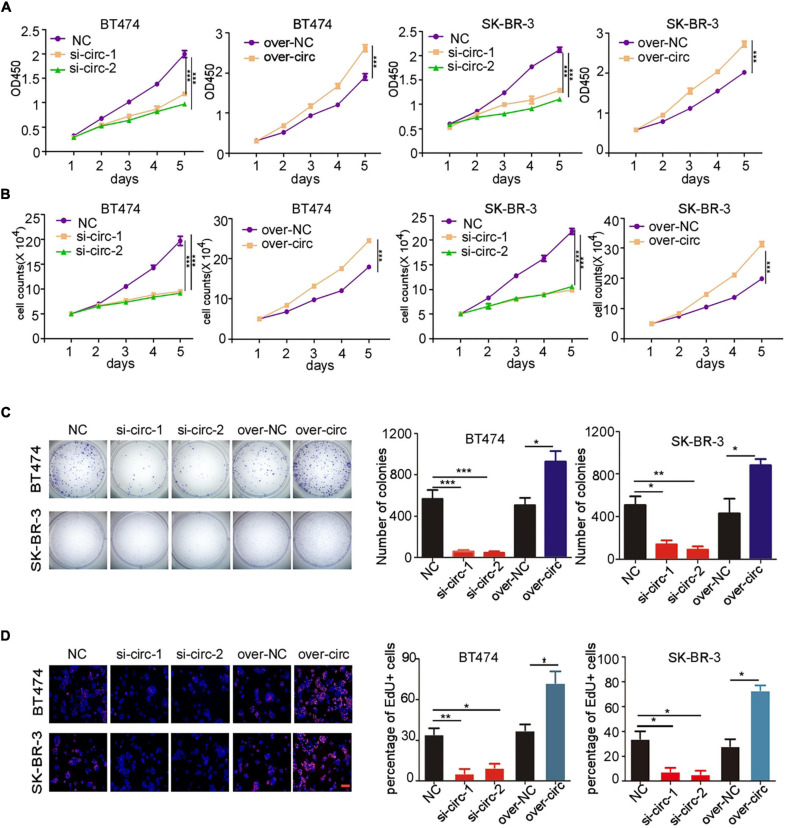
circCDYL promotes proliferation of HER2^+^ BC cells. **(A,B)** The proliferation of SK-BR-3 and BT474 cells after silencing circCDYL or over-expressing circCDYL, as detected by CCK-8 assay **(A)** and cell counting experiments **(B)**. **(C)** Proliferation of SK-BR-3 and BT474 cells, as detected by plate colony formation (left) and quantitative analysis of colonies of each group (right). **(D)** EdU staining in SK-BR-3 and BT474 cells under siRNA or over-expressing plasmid treatment (left) and the percentage of EdU positive cell in each group (right). *NC: negative control*. All experiments above were repeated at least three times. *P < 0.05, **P < 0.01, and ***P < 0.005. Error bars indicate S.E.M.

Our previous study has showed that up-regulation of circCDYL augmented the cell proliferation and autophagy of HER2^–^ BC cells (Liang et al., 2020), but the biological function of circCDYL in HER2^+^ BC cells was not explored. Therefore, we investigated whether circCDYL could modulate the cell proliferation, one of the most important characteristics of cellular fates, in HER2^+^ BC cells. In this study, silencing circCDYL by specific siRNAs or over-expressing circCDYL by plasmids could successfully decrease or increase circCDYL in HER2^+^ BC cells without affecting the expression of CDYL linear RNA, as detected by qRT-PCR (Supplementary Figures 1C,D). Cell proliferation rate of HER2^+^ BC cell lines SK-BR-3 and BT474 was impaired after silencing circCDYL but was augmented after over-expressing circCDYL, as detected by CCK-8 (Figure 2A) and cell-counting assays (Figure 2B). Similarly, plate colony formation and EdU staining experiments also indicated that circCDYL promoted the proliferation of HER2^+^ BC cells (Figures 2C,D). As reported previously, circCDYL promoted the autophagosome formation of HER2^–^ BC cells. However, silencing or over-expression of circCDYL had no effect on the autophagic level of SK-BR-3 in this study (Supplementary Figure 1E). All these suggest that circCDYL does not play similar roles in HER2^+^ and HER2^–^ BC cells, therefore, we next investigated the role of circCDYL in HER2^+^ BC cells. Mechanically, over-expression of circCDYL promoted the activation of PI3K/AKT signal pathway in SK-BR-3 cells (Supplementary Figure 2A). The promoting effect of circCDYL over-expression on proliferation was partly impaired by AKT inhibitor capivasertib (0.5 μM), as detected by CCK8 experiment (Supplementary Figure 2B), indicating that circCDYL promotes proliferation of HER2^–^ BC cells through activating of PI3K/AKT signaling pathway.

### CircCDYL Promotes Tumorigenesis of HER2^+^ BC *in vivo*

**FIGURE 3:**
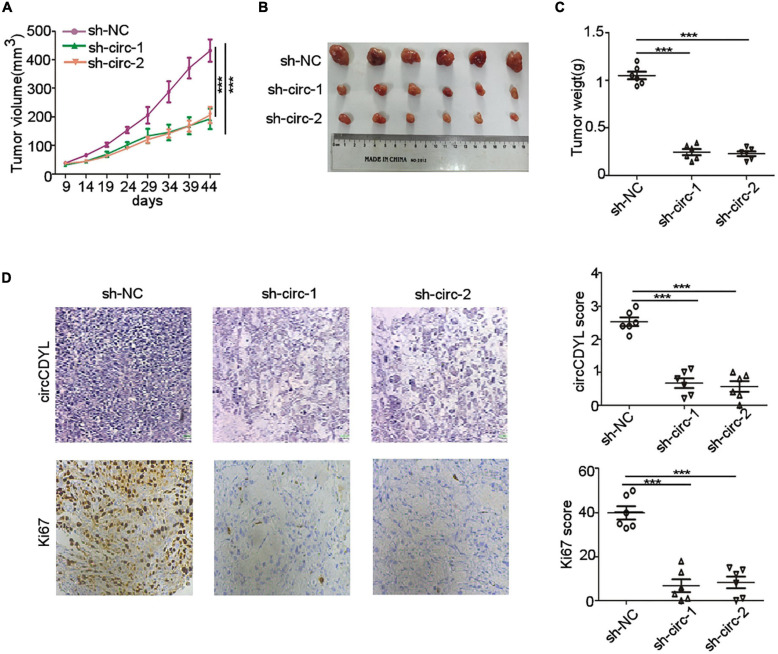
circCDYL promotes progression of HER2^+^ BC *in vivo*. **(A)** Tumor volume derived from SK-BR-3 cells in Balb/c nude mice was measured at various time point. **(B,C)**. Tumor size **(B)** and tumor weight **(C)** was measured after tumor excision. **(D)**. *ISH* detection of circCDYL and IHC staining of Ki-67 in tumors tissues. Scale bar 50 μm. ***P < 0.005. Error bars indicate S.E.M.

To further investigate the biological role of circCDYL in HER2^+^ BC *in vivo*, we established BC orthotopic model in Balb/c nude mice. The tumors derived from SK-BR-3 cells with stable knockdown of circCDYL grew slower than the control group (Figure 3A). The average tumor size of circCDYL knocking-down group was much smaller than NC group by measuring tumor sizes after tumor excision (Figures 3B,C). In addition, the percentage of Ki67 positive cells in circCDYL knocking-down tumors was significantly less than that in NC group (Figure 3D). The results above demonstrate that over-expression of circCDYL promotes the tumorigenesis of HER2^+^ BC *in vivo*.

### CircCDYL Is Degraded in a miR-92b-3p-Dependent Manner

**FIGURE 4:**
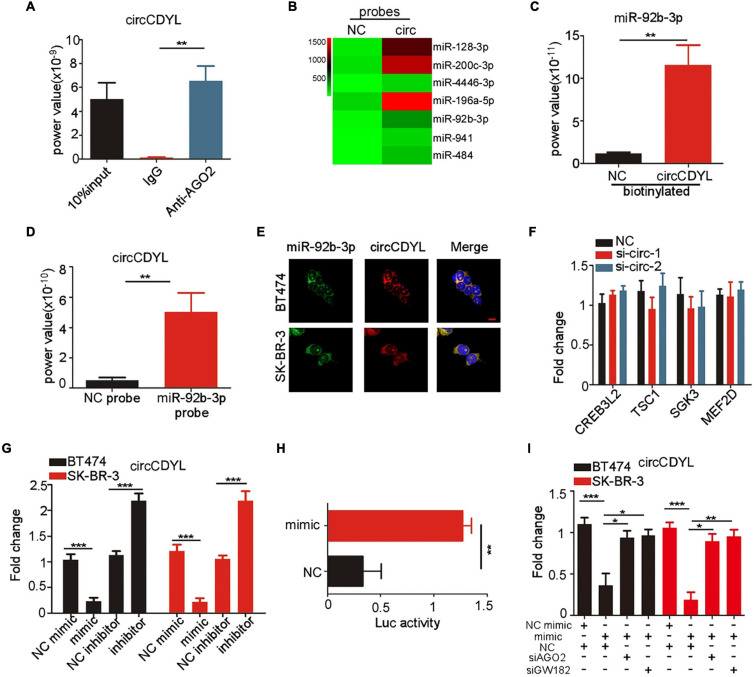
circCDYL is degraded in miR-92b-3p-dependent RISC manner. **(A)** qRT-PCR detection of circCDYL in RNA sample pulled down by AGO2 antibody in RIP experiment. **(B)** MiRNA profile of RNA sample pulled down by circCDYL probes in circRNA pull-down assay, as detected by miRNA deep-sequencing. **(C)** qRT-PCR detection of miR-92b-3p in RNA sample pulled down by circCDYL probes in circRNA pull-down assay. **(D)** qRT-PCR detection of circCDYL in RNA sample pulled down by miR-92b-3p probes in miRNA pull-down assay. **(E)** FISH assay to detect the co-location of circCDYL and miR-92b-3p in BT474 and SK-BR-3 cells. **(F)** qRT-PCR detection of miR-92b-3p targeted genes (CREB3L2, TSC1, SGK3, and MEF2D) in SK-BR-3 cells after silencing circCDYL. **(G)** qRT-PCR detection of circCDYL in SK-BR-3 and BT474 cells after miR-92b-3p mimic or inhibitor transfection. **(H)** Dual luciferase assay in SK-BR-3 cells co-transfected with miR-92b-3p mimic and luciferase reporter plasmid (inserted with full length of circCDYL). **(I)** qRT-PCR detection of circCDYL in SK-BR-3 and BT474 cells after transfection of miR-92b-3p mimic or con-transfection of miR-92b-3p mimic and AGO2 or GW182 siRNA. All experiments above were repeated at least three times. *P < 0.05, **P < 0.01, and ***P < 0.005. Error bars indicate S.E.M.

MiRNA sponge was the most frequently reported mechanism of circRNAs, and circRNAs that can form circRNA-AGO2 complex are reported to have a potential to act as miRNA sponge. The AGO2 RIP experiment was performed in SK-BR-3 cells and showed that circCDYL was abundantly enriched by AGO2 antibody (Figure 4A). In our previous study, circCDYL acted as miR-1275 sponge in HER2^–^ BC cells (MCF7 and MDA-MB-231) (Liang et al., 2020). However, circRNA pull-down experiments in SK-BR-3 indicated that miR-1275 was rarely enriched by circCDYL probes (Supplementary Figure 3A), which might be due to low expression of miR-1275 in HER2^+^ BC cells (Supplementary Figure 3B). To find out circCDYL-binding miRNAs, miRNA deep-sequencing was performed to detect the RNA sample enriched by circCDYL pull-down experiment, and miRNAs candidates that were enriched by 10-folds or over were screened out (Figure 4B). Among the top 5 miRNA candidates, qRT-PCR analysis showed that only miR-92b-3p was verified to enrich by circCDYL probes in circRNA pull-down in SK-BR-3 cells (Figure 4C and Supplementary Figure 3C). Similarly, miRNA pull-down assay indicated that miR-92b-3p probes obviously enriched circCDYL (Figure 4D). By RNA hybrid online database, the 565nt of circCDYL was a strong binding site for miR-92b-3p (Supplementary Figure 3D). Co-location assay by FISH indicated that miR-92b-3p and circCDYL were overlapped in cytoplasm in both SK-BR-3 and BT474 cells (Figure 4E).

It has been reported that CREB3L2, TSC1, SGK3, and MEF2D are downstream genes of miR-92b-3b (Hu et al., 2017; Li T. et al., 2019; Ye et al., 2020; Huang et al., 2021). In HER2^+^ BC cells, we found that these genes were down-regulated after miR-92b-3p mimics transfection in SK-BR-3 cells (Supplementary Figure 3E). However, silencing circCDYL had no effect on expression of CREB3L2, TSC1, SGK3 and MEF2D mRNA in SK-BR-3 cells (Figure 4F), suggesting that circCDYL may not act as miR-92b-3p sponge. Interestingly, circCDYL was down-regulated after miR-92b-3p mimic transfection and up-regulated after miR-92b-3p inhibitor transfection (Figure 4G), while silencing circCDYL had no effect on expression of miR-92b-3p in SK-BR-3 cells (Supplementary Figure 3F). AGO2, as an essential component of RNA-induced silencing complex (RISC), leads to gene silence in a siRNA or miRNA-dependent manner (Marzec, 2020). Therefore, we speculated whether circCDYL could be silenced in a miR-92b-3p dependent RISC manner. After silencing AGO2 or GW182 (another component in RISC complex) in SK-BR-3, expression of circCDYL was up-regulated significantly (Supplementary Figure 3G), suggesting that circCDYL was degraded in a RISC manner. In addition, a dual luciferase reporter (inserted full length of circCDYL) assay was performed, and miR-92b-3p mimic reduced the luciferase reporter activity by 68% (Figure 4H). While silencing either AGO2 or GW182 in SK-BR-3 cells, miR-92b-3p mimic transfection exhibited slight effect on circCDYL expression (Figure 4I), indicating circCDYL is silenced in a miR-92b-3p dependent RISC manner in HER2^+^ cell.

Interestingly, miR-92b-3p mimic transfection decreased the expression of circCDYL in HER2^–^ BC cells (MCF-7 and MDA-MB-231 cells) as well. When silencing either AGO2 or GW182 in MCF-7 and MDA-MB-231 cells, miR-92b-3p mimic transfection exhibited slight effect on circCDYL expression (Supplementary Figure 4A), indicating that miR-92b-3p- dependent RISC manner in degradation of circCDYL existed in HER2^–^ BC cells as well as in HER2^+^ BC cells. We compared the expression of miR-92b-3p in HER2^–^ and HER2^+^ BC cells, and miR-92b-3p was obviously higher expressed in HER2^+^ BC cells than that in HER2^–^ BC cells (Supplementary Figure 4B), indicating that miR-92b-3p- dependent RISCI manner on circCDYL may exhibit more effect in HER2^+^ BC cells than in HER2^–^ BC cells.

### MiR-92b-3p Inhibits Cell Proliferation of HER2^+^ BC Cells by Silencing circCDYL

**FIGURE 5:**
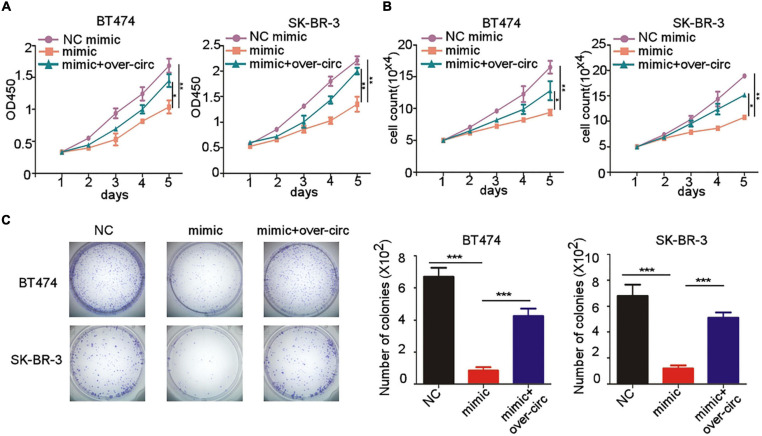
miR-92b-3p inhibits proliferation of HER2^+^ BC cells by down-regulation of circCDYL. **(A,B)** The proliferation of SK-BR-3 and BT474 cells after transfection of miR-92b-3p mimic or co-transfection of miR-92b-3p mimic and circCDYL over-expressing plasmid, as detected by CCK-8 assay **(A)** and cell counting experiments **(B)**. **(C)** Plate colony formation to detect the proliferation of SK-BR-3 and BT474 cells after transfection of miR-92b-3p mimic or co-transfection of miR-92b-3p mimic and circCDYL over-expressing plasmid (left), and quantitative analysis of colonies of each group (right). *P < 0.05, **P < 0.01, and ***P < 0.005. Error bars indicate S.E.M.

Since the function of miR-92b-3p in BC was still unclear, we next investigated whether miR-92b-3p was involved in cell proliferation through regulation of circCDYL. CCK-8 assay showed that miR-92b-3p mimic transfection impaired proliferation rate of SK-BR-3 and BT474 cells (Figure 5A). The inhibitory effect of miR-92b-3p mimic in HER2^+^ BC cells was partly rescued by over-expression of circCDYL (Figure 5A). The result of cell-counting assays and plate colony formation assay drew the similar conclusion (Figures 5B,C), indicating miR-92b-3p inhibits cell proliferation of HER2^+^ BC cells *via* circCDYL degradation.

### CircCDYL and miR-92b-3p Expression Correlates With Clinical Outcome of HER2^+^ BC Patients

**FIGURE 6:**
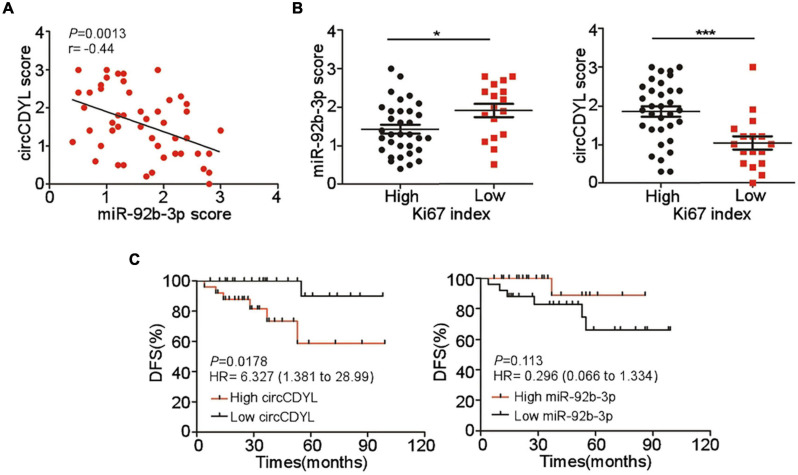
Clinical significance of circCDYL and miR-92b-3p in HER2^+^ BC. **(A)** The correlations between the expression of circCDYL and miR-92b-3p, as detected by ISH in 50 HER2^+^ BC patient. **(B)** The expression of miR-92b-3p and circCDYL in HER2^+^ BC patients with high Ki67 index (> 14%) and low Ki67 index (< = 14%). **(C)** Kaplan–Meier analysis of Disease-free survival of HER2^+^ BC patients (*n* = 50) with high/low expression of circCDYL (left) or miR-92b-3p (right).

Expression of circCDYL and miR-92b-3p in tumor sections of 50 HER2^+^ BC patients was detected by *ISH*. Pearson correlation analysis showed that miR-92b-3p expression was negatively correlated with circCDYL level (Figure 6A). We further analyzed the relationship between the proliferative marker Ki67 and expression of circCDYL or miR-92b-3p, and found that the tumor with higher Ki67 index tended to have a lower expression of miR-92b-3p and higher expression of circCDYL (Figure 6B). Importantly, Kaplan–Meier analysis indicated that HER2^+^ BC patients with higher expression of circCDYL exhibited a poorer disease-free survival (DFS) (HR = 6.327, *P* = 0.0178) (Figure 6C). Though patients with low expression of miR-92b-3p showed poorer DFS as well, the statistical significance was not found between miR-92b-3p low group and high group (HR = 0.296, *P* = 0.113) (Figure 6C).

## Discussion

Breast cancer (BC) is the most common malignant tumor and leading cause of death by tumor among female worldwide (Bray et al., 2018). Among these BC cases, around 15-20% is HER2^+^ subtype, and the positive status of HER2 indicates poorer clinical outcome of BC patients. Tough numerous researches reveal the mechanism of HER2^+^ BC progression, the underlying mechanisms, especially non-coding RNA, in progression of HER2^+^ BC patients are not clear. Our previous study identified an autophagy-associated circRNA circCDYL, which promoted cell proliferation and autophagosome formation of HER2^–^ BC cells *via* miR-1275 sponge. However, the role of circCDYL in regulating cellular fate of HER2^+^ BC cells was not elucidated (Liang et al., 2020). Biological functional experiments proved that circCDYL promoted the proliferation of HER2^+^ BC cells, but had no influence on autophagic level. Mechanism study indicated that circCDYL did not act as miR-1275 sponge in HER2^+^ BC cells, as miR-1275 rarely expressed in HER2^+^ BC cell lines.

The relationship between circCDYL and HER2 gene was further investigated. CircCDYL was commonly high-expressed in BC, no matter the statue of HER2 gene. Knocking down HER2 gene in HER2^+^ BC cells and over-expressing HER2 gene in HER2^–^ BC cell lines did not change the expression of circCDYL, indicating that HER2 gene did not regulate the expression of circCDYL. Though circCDYL promoted proliferation of both HER2^+^ and HER2^–^ BC cells, several mechanism of circCDYL was specific in HER2^+^ BC. Firstly, circCDYL promoted progression of BC mainly *via* miR-1275-ATG7/ULK1-autophagy axic in HER2^–^ BC (Liang et al., 2020), while circCDYL promoted progression of HER2^+^ BC *via* activation of PI3K-AKT pathway. Secondly, the manner of miR-92b-3p-dependent cleavage on circCDYL was specific in HER2^+^ BC cells, as miR-92b-3p rarely expressed in HER2- BC cells.

In addition, several interesting findings in mechanism of circRNA were reported in this study. Firstly, published evidences shows that the circRNAs act as miRNA sponge if these circRNAs can form AGO2-circRNA complex (Huang et al., 2019; Liang et al., 2020; Zhou C. et al., 2020). In this study, we found that circCDYL could form AGO2-circCDYL complex, and could strongly interact with miR-92b-3p in HER2^+^ BC cells. However, circCDYL had no influence on expression of miR-92b-3p targeted genes, indicating that circCDYL did not act as miR-92b-3p sponge in HER2^+^ BC cell lines. The results above suggested that AGO2-circRNA complex was not a gold standard to reveal miRNA sponge potential of circRNAs. Secondly, AGO2 is an important component of RISC complex, and RISC complex exhibits nuclease function in degradation of RNA (Marzec, 2020). Interestingly, miR-92b-3p mimic transfection resulted in down-regulation of circCDYL in both HER2^+^ and HER2^–^ BC cells, and AGO2 silencing could almost prevent the down-regulation of circCDYL from miR-92b-3p mimic transfection, indicating that circCDYL was degraded in a miR-92b-3p dependent RISC manner. Several researches have discussed about the degradation of circRNA. For example, RNase L was reported to degrade circRNAs when the cell was infected by virus (Liu et al., 2019), and structure-mediated RNA decay induced by UPF1 and G3BP1 protein was important pathway for degradation of circRNAs (Fischer et al., 2020). Our study provides a new mechanism of circRNA degradation that miRNA-dependent RISC manner is an alternative way for cleanup of circRNAs in eukaryotic cells. Thirdly, circCDYL acted as miR-1275 sponge in HER2^–^ BC cells (Liang et al., 2020), but did not act the same way in HER2^+^ BC cell, as miR-1275 was rarely expressed in HER2^–^ BC cell lines. These findings indicated that mechanism of circRNAs was various in different subtypes of breast cancer, and mechanism of circRNA was dependent on the expression of downstream genes.

The role of miR-92b-3p in cancer is contentious. MiR-92b-3p exhibited tumor-promoting role in proliferation, migration and invasion of colorectal, renal, gastric and prostate cancer cells (Gong et al., 2018; Li C. et al., 2019; Wang et al., 2020; Wang G. et al., 2021). On the contrary, miR-92b-3p suppresses tumor progression of pancreatic cancer by targeting Gabra3 (Long et al., 2017). However, the role of miR-92b-3p is unclear in BC, and this is the first study to reveal the biological function of miR-92b-3p in BC. In this study, we found that MiR-92b-3p acted as a tumor-repressive miRNA and inhibited cell proliferation of BC cells by down-regulation of circCDYL.

Breast cancer is a subtype with poor clinical outcome, and identifying new biomarkers for predicting prognosis of BC patients is of tremendous clinical significance. CircRNA and miRNA are proved to be stable and abundant, and exhibits disease-specific characteristic in eukaryotic cells, for which both of them are considered to be ideal biomarkers for human diseases (Jahani et al., 2020; Galvão-Lima et al., 2021). In this study, we identified the stability of circCDYL in HER2^+^ BC cells. CircCDYL was up-regulated in tumor tissues of HER2^+^ BC patients when comparing with the adjacent normal tissues. Importantly, high expression of circCDYL or low expression of miR-92b-3p was associated with poor disease-free survival of HER2^+^ BC patients. Therefore, our finding provided two potential biomarkers for HER2^+^ BC patients. For the patients with high expression of circCDYL or low expression of miR-92b-3p, more aggressive treatment or closer follow-up should be considered. CircCDYL exhibited powerful biological function in regulating cellular fate in both HER2^+^ and HER2^–^ BC cells, for which circCDYL might be a potential therapeutic target for BC patients.

In this study, circCDYL was proved to promote proliferation of HER2^+^ BC cells *via* the activation of PI3K/AKT signal pathway, however, the downstream mechanism how circCDYL promoted activation of PI3K/AKT signal pathway remain largely unknown. In this research, no other miRNAs except miR-92b-3p was found to interact with circRNAs, and we had no evidence to prove the miRNA sponge potential of circCDYL in HER2^+^ BC cells. In addition, circCDYL mainly located in the cytoplasm of HER2^+^ BC cell and could not promote transcription of gene by interacting with pol-II protein. Moreover, circCDYL exhibited little possibility to be a protein translation template, for the reason that circCDYL did not have open reading frame (ORF) and internal ribosome entry site (IREs), as predicted by circRNAdb online database. Enormous studies determine that circRNAs can regulate biological function of cancer cells by interacting with protein (Du et al., 2016, 2018; Sun et al., 2019). CircCDYL might promote cell proliferation of HER2^+^ BC cells by interacting with certain protein, which involve in the activation of PI3K/AKT pathway.

## Data Availability Statement

The datasets presented in this study can be found in online repositories. The names of the repository/repositories and accession number(s) can be found below: https://www.ncbi.nlm.nih.gov/geo/query/acc.cgi?acc=GSE174541, accession number: GSE174541.

## Ethics Statement

The studies involving human participants were reviewed and approved by Sun Yat-sen Memorial Hospital, Sun Yat-sen University. The patients/participants provided their written informed consent to participate in this study. The animal study was reviewed and approved by Forevergen Medical Corporation (Guangzhou, China), and all procedures were in accordance with the ethical guidelines of the institution.

## Author Contributions

GL and YL contributed to the acquisition, analysis, and interpretation of the data and drafting of the manuscript. QL, YS, QL, YC, MM, and AH contributed to the data collections and manuscript review and drafting of the manuscript. CG and JL supervised the study. All authors read and approved thefinal manuscript.

## Conflict of Interest

The authors declare that the research was conducted in the absence of any commercial or financial relationships that could be construed as a potential conflict of interest.

## Publisher’s Note

All claims expressed in this article are solely those of the authors and do not necessarily represent those of their affiliated organizations, or those of the publisher, the editors and the reviewers. Any product that may be evaluated in this article, or claim that may be made by its manufacturer, is not guaranteed or endorsed by the publisher.
