# Supplementary material for: MiR-92b-3p Inhibits Proliferation of HER2-Positive Breast Cancer Cell by Targeting circCDYL
Source: Front Cell Dev Biol. 2021 Jul 29;9:707049. doi: 10.3389/fcell.2021.707049 (PMC8358302; doi:10.3389/fcell.2021.707049)
Supplement: Supplementary file 1 [file Data_Sheet_1.pdf]

Title: MiR-92b-3p inhibits proliferation of HER2-positive breast cancer cell by targeting circCDYL.

Authors: Gehao Liang[1,2,#], Yun Ling[1,3,#], Qun Lin[1], Yu Shi[1], Qing Luo[1], Yinghuan Cen[1], Maryam Mehrpour[5], Ahmed Hamai[5], Jun Li[4*], Chang Gong[1*]

[1]Breast Tumor Center, Sun Yat-sen Memorial Hospital, Sun Yat-sen University, Guangzhou, China.

[2]Department of Breast Oncology, Sun Yat-sen University Cancer Center, Sun Yat-sen University, Guangzhou, China.

[3]Department of Breast Surgery, the Second Affiliated Hospital, Guangzhou Medical University, Guangzhou, China.

[4]Department of Biochemistry, Zhongshan School of Medicine, Sun Yat-sen University, Guangzhou, China.

[5]Institut Necker-Enfants Malades (INEM), Inserm U1151-CNRS UMR 8253, Paris, 75993, France;

[#] These two authors contribute equally to this article.
*Correspondence: Chang Gong, gchang@mail.sysu.edu.cn; Jun Li, lijun37@mail.sysu.edu.cn .

Keywords: miR-92b-3p, RNA induced silencing complex, circCDYL, cell proliferation, HER2-positive breast cancer.

Content: Supplementary Figures *2, Supplementary Tables *3

Supplementary Figure and Figure Legend:

Figure S1

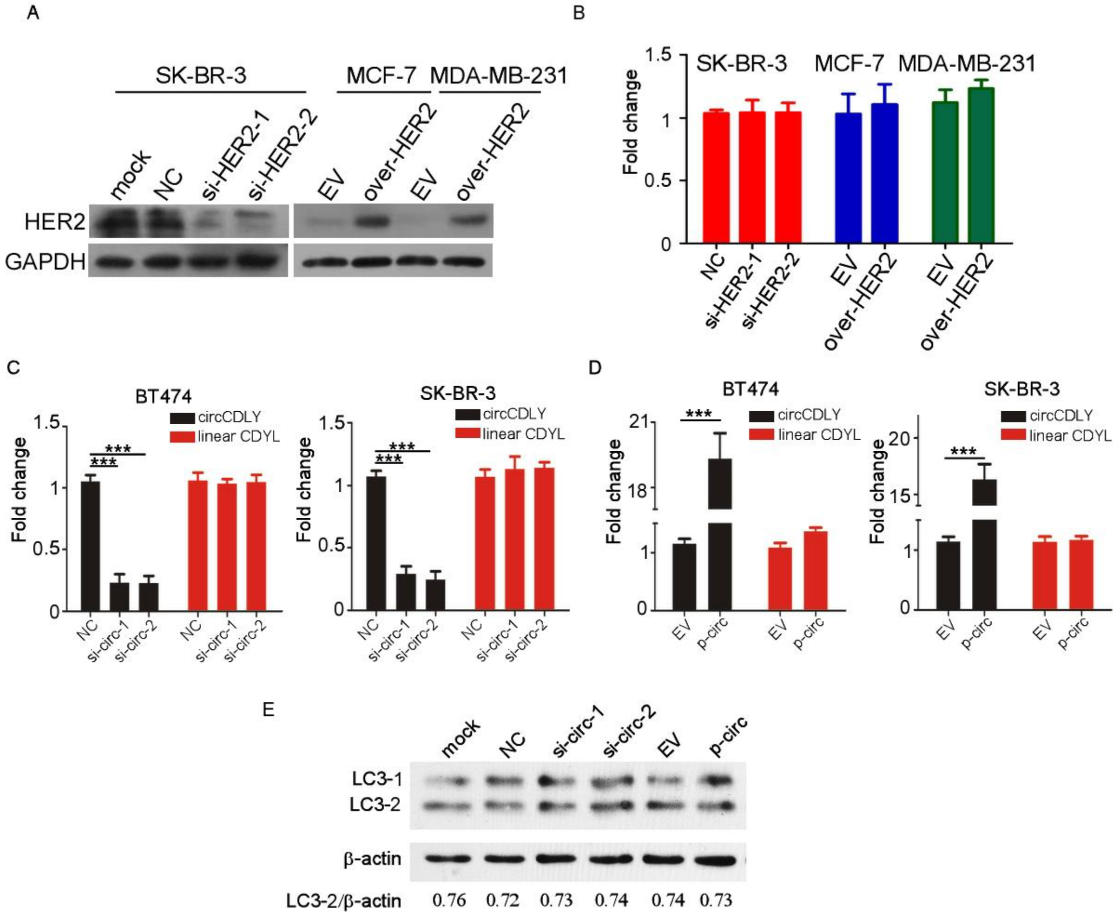

Fig S1. The efficiency of circCDYL siRNAs and over-expressing plasmids in HER2+ BC cells. A. Western Blot detection of HER2 gene in $HER2^+$ BC cells (SK-BR-3) after silencing HER2 gene and in $HER2^-$ BC cells (MCF-7 and MDA-MB-231) after over-expression of HER2 gene. **B.** qRT-PCR detection of circCDYL in SK-BR-3 after silencing HER2 gene and in MCF-7 and MDA-MB-231 after over-expression of HER2 gene. **C.** qRT-PCR detection of circCDYL and linear CDYL in SK-BR-3 and BT464 cells after circCDYL siRNA transfection. **D.** qRT-PCR detection of circCDYL and linear CDYL in SK-BR-3 and BT464 cells after circCDYL over-expressing plasmid transfection. **E.** Western Blot detection of autophagic marker LC3-2 in SK-BR-3 cells after circCDYL knock-down or over-expression.

Figure S2

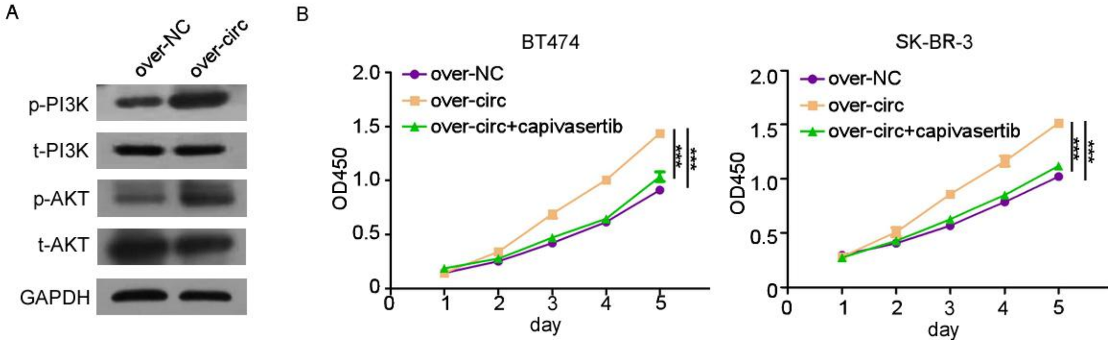

Fig S2. CircCDYL promoted proliferation of HER2$^+$ BC cell via PI3K/AKT pathway signaling. A. Western Blot detection of PI3K/AKT signal pathway after circCDYL over-expression in SK-BR-3 cells. **B.** The proliferation of SK-BR-3 and BT474 cells after treatment with circCDYL over-expressing plasmids or co-treated with circCDYL over-expressing plasmids and AKT inhibitor capivasertib (0.5μM), as detected by CCK-8 assay.

Figure S3

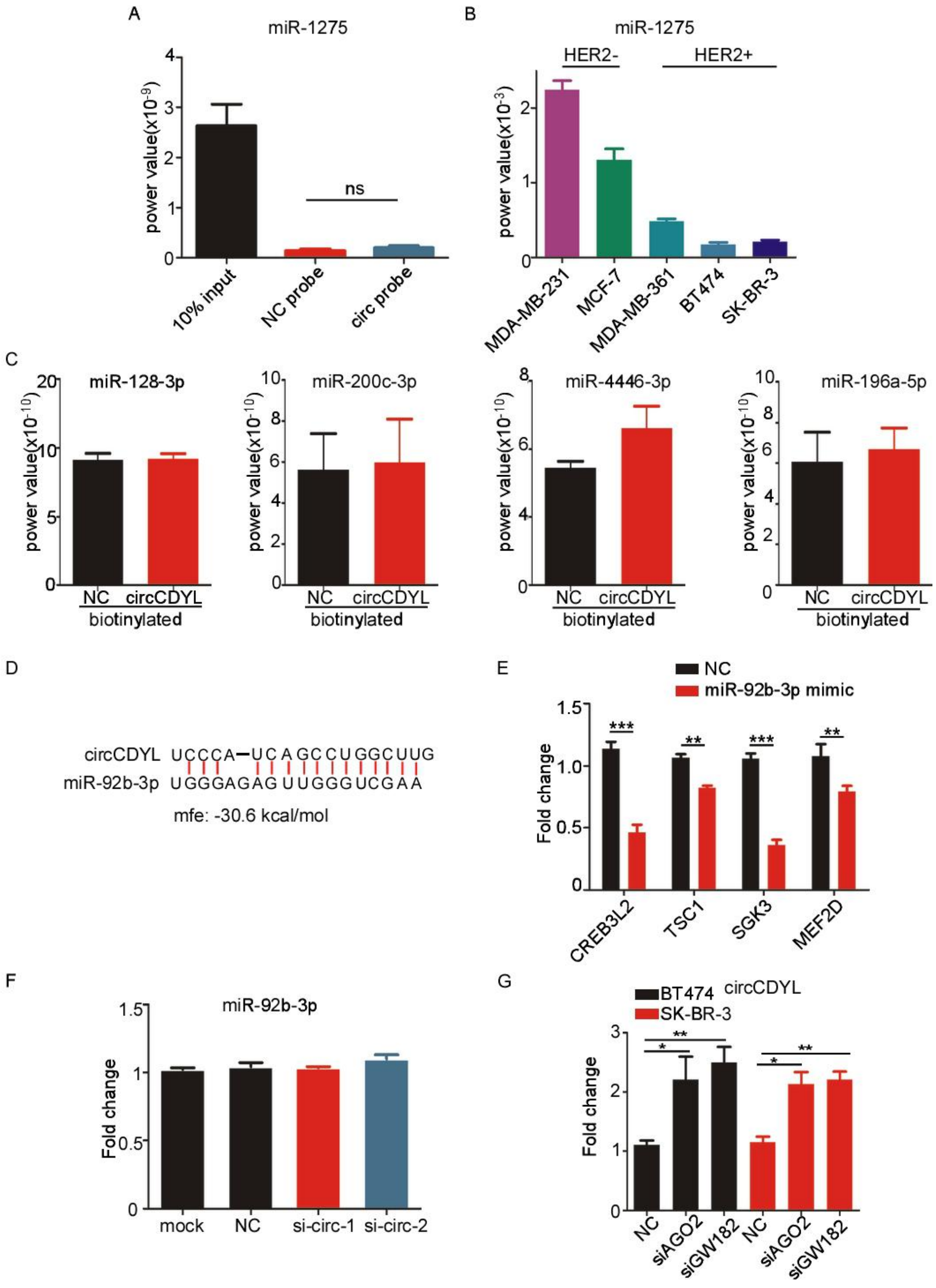

Fig S3. circCDYL was degraded in a miR-92b-3p dependent RISC manner in HER2$^+$ BC cells. A. qRT-PCR detection of miR-1275 in RNA sample pulled down by circCDYL probes in circRNA pull-down assay in SK-BR-3 cells. **B.** qRT-PCR detection of miR-1275 in HER2$^-$ BC cell lines (MDA-MB-231, MCF-7) and HER2$^+$ BC cell lines (MDA-MB-361, BT474, SK-BR-3). **C.** qRT-PCR detection of miR-

128-3p, miR-200c-3p, miR-4446-3p and miR-196a-5p in RNA sample pulled down by circCDYL probes in circRNA pull-down assay in SK-BR-3 cells. **D.** miR-92b-3p binding site in circCDYL, as predicted by RNAhybrid online database. **E.** qRT-PCR detection of CREB3L2, TSC1, SGK3 and MEF2D in SK-BR-3 cells after mR-92b-3p mimic transfection. **F.** qRT-PCR detection of mR-92b-3p in SK-BR-3 cells after circCDYL siRNA transfection. **G.** qRT-PCR detection of circCDYL in SK-BR-3 cells after AGO2 and GW182 siRNA transfection.

Figure S4

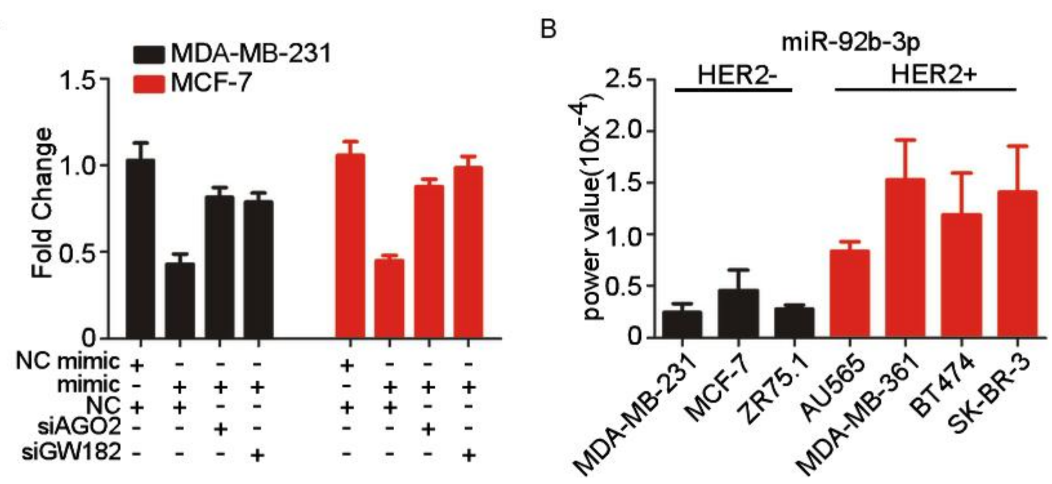

Fig S4. circCDYL was degraded in a miR-92b-3p dependent RISC mannerin HER2$^-$ BC cells. **A.** qRT-PCR detection of circCDYL in SK-BR-3 and BT474 cells after transfection of miR-92b-3p mimic or con-transfection of miR-92b-3p mimic and AGO2 siRNA. **B.** Expression of miR-92b-3p in HER2$^-$ BC cell lines (MCF-7, MDA-MB-231, ZR75-1) and HER2$^+$ BC cell lines (AU565, MDA-MB-361, SK-BR-3, BT474), as detected by qRT-PCR.All experiments above were repeated at least 3 times. *P< 0.05, **P< 0.01, ***P< 0.005. Error bars indicate S.E.M.

Supplementary Table

Table S1. Sequence of siRNA or shRNA used in current stud

Gene	No	Sense (5' --3')	Antisense (5' --3')
circCDYL	si/sh-1	GUUAACGGGAAAGGUUGAAdTdT	UUCAACCUUUCCCGUUAACdTdT
	si/sh-2	ACGGGAAAGGUUGAAAGGAUUdTdT	AAUCCUUUCAACCUUUCCCGTdTdT
AGO2 (mixture of si-1 and si-2)	si-1	GCACGGAAGUCCAUCUGAAdTdT	GCAGGACAAAGAUGUAUUAdTdT
	si-2	GGGUCUGUGGUGAUAAAUAdTdT	GUAUGAGAACCCAAUGUCAdTdT
GW182 (mixture of si-1 and si-2)	si-1	AACACCUUAACAGCAGAUCdTdT	UAGCGGACCAGAGCAUUUCdTdT
	si-2	UAAUCACCAAUAUUUAGGCdTdT	UAGGGCAAGUCCAUUGUUCdTdT

Table S2. Primers used in current study

Gene	Forward primer (5' --3')	Reverse primer (5' --3')
circCDYL	ACCCACTAGTGCCTCAGGTG	TGTCGTCCTCGCTGTCATAG
Linear CDYL	ATTGCCTCTTTGCGATGT	TGTCAGCTTCCGTCCACT
CREB3L2	ACCACACGCACTTCTCAGAAC	GAGGAAAGGATCATTCAGGAGC
TSC1	CAACAAGCAAATGTCGGGGAG	CATAGGGCCACGGTCAGAA
SGK3	GCCCAAGTGTAAGCATTCCCA	GTTTAGTCCTGCTCGTCTTTGTT
MEF2D	CCAGCGAATCACCGACGAG	GCAGTCACATAGCACGCTC
β-actin	TCATGAAGTGTGACGTGGACATC	CAGGAGGAGCAATGATCTTGATCT
GAPDH	AGGTGAAGGTCGGAGTCAAC	CGCTCCTGGAAGATGGTGAT

Table S3. Probes used in current study

Gene	FISH or ISH (5' --3')	Pull down (5' --3')
MiR-92b-3p		Patent of Synbio Company
circCDYL	CAATCCTTTCAACCTTTCCCGTTAAC	CAATCCTTTCAACCTTTCCCGTTAAC
NC	CCAGTGAATCCGTAATCATG	CCAGTGAATCCGTAATCATG

NC, Negative control
